# Dynamic changes of endogenous phytohormones and carbohydrates during spontaneous morphogenesis of *Centaurium erythraea* Rafn

**DOI:** 10.3389/fpls.2024.1487897

**Published:** 2024-11-06

**Authors:** Milana Trifunović-Momčilov, Václav Motyka, Marija Marković, Marija Milovančević, Biljana Filipović, Petre I. Dobrev, Angelina Subotić

**Affiliations:** ^1^ Department for Plant Physiology, Institute for Biological Research “Siniša Stanković” – National Institute of Republic of Serbia, University of Belgrade, Belgrade, Serbia; ^2^ Institute of Experimental Botany of the Czech Academy of Sciences, Prague, Czechia

**Keywords:** centaury, cytokinin, auxin, soluble sugars, morphogenesis, phytohormone

## Abstract

Common centaury (*Centaurium eryhtraea* Rafn) is a medicinal plant species with vigorous morphogenic potential *in vitro*. The process of spontaneous shoot regeneration in a solid root culture is characteristic for this plant species. In this context, the aim of this work was to investigate the dynamic changes of endogenous phytohormones and carbohydrates content in root explants at different time points (0, 2, 4, 7, 14, 21, 28, and 60 days) during spontaneous centaury morphogenesis *in vitro*. Detailed analysis of cytokinins (CKs) showed that *trans*-zeatin (*tZ*) was the major bioactive CK at all time points. The corresponding riboside, *t*Z9R, was also determined in the majority of the identified transport forms, at all time-points. Further analysis of endogenous auxin revealed a significant increase in endogenous indole-3-acetic acid (IAA) after 21 days, when a huge jump in the ratio of IAA/bioactive CKs was also observed. The maximum total soluble sugar content was measured after 14 days, while a significant decrease was determined after 21 days, when the first regenerated adventitious shoots appeared. This undoubtedly indicates an increased energy requirement prior to the actual regeneration of the shoots. The obtained results indicate that the period from day 14 to day 21 involves the most dramatic disturbances in endogenous bioactive CKs, IAA and carbohydrate balance, which are very important and valuable factors for the onset of shoot regeneration.

## 1 Introduction

The growth and development of plants depend on nutrients, hormones and their interactions, but are also influenced by various environmental factors. Plant hormones, naturally occurring substances with diverse chemical compositions and structures, affect plant growth in response to external environmental stimuli. The concentrations and activity of hormones depend on biosynthesis, transport, conjugation, accumulation and degradation in the plant cell (Bajguz and Piotrowska-Niczyporuk, 2023).

Cytokinins (CKs) represent a group of plant hormones involved in numerous developmental processes in plants such as the promotion of shoot growth by cell proliferation of apical and axillary meristematic cells, the inhibition of root growth by promoting cell differentiation in the apical meristem, the development of vascular and cambial tissue, the delay of leaf senescence, and seed germination (Kieber and Schaller, 2018). According to their chemical structure, all naturally occurring CKs are adenine derivatives. Although CKs are synthesized in the shoot meristem and in young leaves, the most important organ for the biosynthesis of adenine-type CKs is the plant root. Since CKs are also found in the xylem sap, they are considered one of the most important messengers transported over long distance between roots and shoots (Sakakibara, 2021). The biosynthesis and degradation pathways of CKs have been largely elucidated and the genes responsible for these metabolic pathways have mostly been described. The biosynthesis of CK depends mainly on the activity of adenosine phosphate-isopentenyl transferase, which transfers the isopentenyl group to an *N^6^*-(Δ^2^-isopentenyl) adenine (iP) nucleotide and further to *trans*-zeatin (*t*Z), the most abundant form of CKs in plants (Haberer and Kieber, 2002). On the other hand, CKs can be reversibly inactivated by the glucosylation or irreversibly degraded by cytokinin oxidase/dehydrogenase (Hirayama and Mochida, 2022).

In addition to CKs, auxins are involved in plant growth and development of all organs, especially in the early stages. Young leaves and shoot apical meristem are the main sites where auxin is produced and transported to the root via the phloem (Ljung, 2013). The most bioactive naturally occurring auxin form in plants is indole-3-acetic acid (IAA). The tryptamine pathway is one of the four tryptophan-dependent pathways of IAA biosynthesis in plants. In this pathway tryptophan is first converted to tryptamine and then converted to indole-3-acetaldehyde (I3A) and finally to IAA (Tang et al., 2023). On the other hand, the oxidation of IAA to 2-oxindole-3-acetic acid (oxIAA) is the major catabolic pathway that regulates IAA levels in plants (Zhang and Peer, 2017). Further conversion of oxIAA into various conjugates such as di- oxIAA, oxIAA-Asp, and oxIAA-Glu leads to irreversible IAA degradation. As CKs, auxin conjugates function as storage forms, intermediates in degradative processes or protection against oxidative degradation (Zhao, 2014). In general, auxin conjugates, including ester-linked simple and complex carbohydrate conjugates, amide-linked amino acid conjugates, amide-linked peptide and protein conjugates, are considered inactive auxin forms (Korasick et al., 2013). The amide-linked conjugates such as IAA-aspartate (IAA-Asp) and IAA-glutamate (IAA-Glu) are classified as catabolites because they cannot be hydrolyzed to IAA. These irreversible conjugates are much more abundant in plants than the reversible ones (Casanova-Sáez et al., 2021). Additionally, ester-linked IAA-conjugates serve as auxin storage forms than can be reversibly converted to free IAA and thus contribute to the maintenance of the bioactive auxin pool (Korasick et al., 2013).

The effect of plant hormones during different developmental processes depends on relative concentrations, signal transduction and biosynthetic pathways. In general, CKs and auxin are considered antagonistic hormones. The essential role of CKs and auxin has also been established in shoot regeneration *in vitro*, on explants originated from different plant organs. In various plant species, shoot organogenesis can occur directly on the surface of the explants or indirectly – via the callus stage (Raspor et al., 2021). In practice, shoot organogenesis *in vitro* is usually induced by the application of different hormones and it is obvious that these exogenous substances trigger a complex cellular and genetic network of morphogenesis. However, in rare plant species spontaneous shoot regeneration can also be observed. In this case, it can be concluded that the level of the individual endogenous hormones and their crosstalk are crucial and relevant for the specific physiological state of the plant tissue.

Beside plant hormones, plant growth and development also depend on sugars, as they are the primary source of carbon and energy for the cells. Sugars act as effective signaling molecules and as direct substrates for intermediary metabolism. As signaling substances, sugars affect every aspect of plant life, from seed germination and further vegetative phase to reproductive phase and seed formation (Dahiya et al., 2017). In plant cell, tissue and organ culture 3% sucrose is the most commonly used carbon source. The presence of a high sucrose concentration reduces photosynthesis and promotes structural and physiological changes that lead to stomata closure (Kottapalli et al., 2018). In tissue culture media, sucrose provides energy for all metabolic processes and maintains the osmotic potential. During morphogenesis, exogenous carbohydrates have very distinct effects on organogenesis, and their individual impact at different growth stages is specific to each individual species (Yaseen et al., 2013). On the other hand, the metabolism of endogenous soluble carbohydrates during *in vitro* shoot development is not well understand yet. Nevertheless, important progress has been made in the last decade with various approaches to decipher and identify the broad range of functions and effects of sugars and sugar-derived metabolic signals on the regulatory systems controlling plant growth. Guo et al. (2023) found that these systems, which are influenced by different sugars, could be either growth-promoting or growth-inhibiting.

Common centaury (*Centaurium erythraea* Rafn) is a plant species that attracts attention due to its numerous medicinal properties, but also due to the strong morphogenic potential of root and shoot explants (Simonović et al., 2021). The process of spontaneous shoot regeneration in a solid root culture is characteristic of this plant species. Almost 20 years ago, histological studies revealed that spontaneously regenerated adventitious buds originate from meristematic cells derived from the root cortex tissues (Subotić et al., 2006). The above-mentioned growth and development regulators, hormones and sugars, play a predominant role and act synergistically, antagonistically and sometimes independently to trigger the final plant response. Considering that most of the previous knowledge on *in vitro* morphogenesis comes from the analysis of plant tissue cultured in the presence of exogenously applied hormones, the aim of this work was to evaluate the dynamic changes of endogenous phytohormones and soluble sugars content in order to elucidate and better understand the process of spontaneous morphogenesis in centaury.

## 2 Materials and methods

### 2.1 Plant material, culture conditions and experimental design

*C. erythraea* plants were initiated and maintained as previously described by Trifunović-Momčilov et al. (2016). Three-month-old mother stock cultures were selected as primary plant material. To establish a root culture, root explants (≈ 10 mm long) were cut off from the mother stock plants and then transferred to half-strength hormone-free MS medium (½MS, Murashige and Skoog, 1962) solidified with 0.7% agar and supplemented with 3% sucrose and 100 mg l^-1^ *myo*-inositol. The medium was adjusted to pH 5.8 with NaOH/HCl and autoclaved at 121°C for 25 minutes. All *in vitro* cultures were grown at 25 ± 2°C and a 16/8h light/dark photoperiod (“Tesla” white fluorescent lamps, 65W, 4500K; light flux of 47 μmol s^-1^ m^-2^). The excised root explants were further cultured on ½MS medium at eight different time points (0, 2, 4, 7, 14, 21, 28 and 60 days). The samples were collected at each time point, immediately frozen in liquid nitrogen and stored at -80°C until further analysis.

### 2.2 Extraction, purification and quantification of endogenous phytohormones

Phytohormones were extracted and quantified according to Prerostova et al. (2021). Samples were homogenized with 1.5 mm zirconium beads using a FastPrep-24 instrument (MP Biomedicals, CA, USA) with 100 µl 1M HCOOH in water and a mixture of internal standards. After centrifugation at 17 500 rpm for 20 min and re-extraction, the combined supernatant was applied to SPE Oasis HLB 10mg 96-well plate (Waters, Milford, MA, USA). The supernatant was forced through the SPE plate using the positive pressure manifold Pressure+96 (Biotage, Uppsala, Sweden). The SPE 96-well plate was washed three times with 100 µl water. The samples were eluted with 100 ul 50% acetonitrile/water (v/v). An aliquot of 5 µl of the SPE eluate was injected into the LCMS system. The phytohormones were separated on Kinetex EVO C18 column (2.6 µm, 150 x 2.1 mm, Phenomenex, Torrance, CA, USA). The mobile phases consisted of A) 5 mM ammonium acetate and 2 µM medronic acid in water, and B) 95/5 acetonitrile/water (v/v). The following gradient program was applied: 5% B in 0 min, 7% B in 0.1 to 5 min, 10% to 35% in 5.1 to 12 min, 100% B at 13 to 14 min, and 5% B at 14.1 min. Phytohormone analysis was carried out using an LC-MS system consisting of UHPLC 1290 Infinity II (Agilent, Santa Clara, CA, USA) coupled to 6495 Triple Quadrupole mass spectrometer (Agilent). MS analysis was performed in MRM mode, using the isotope dilution method. Data acquisition and processing were performed using Mass Hunter software B.08 (Agilent). The amount of each phytohormone analysed is expressed as pmol/g of the sample fresh weight (FW).

### 2.3 Quantification of total soluble sugars

The concentration of total soluble sugar (TSS) was measured using the traditional anthrone colorimetric method (Le and Stuckey, 2016). The plant material (100 mg) was homogenized in liquid nitrogen and 2.5 N HCl. The homogenate was boiled for 3 hours, and after cooling, Na_2_CO_3_ was added for neutralization. The material was then centrifuged for 2 minutes, and the supernatant was mixed with 0.2% anthrone (Sigma Aldrich, St. Louis, MO, USA) dissolved in concentrated sulfuric acid and boiled for 10 min. After cooling, the TSS concentration was determined by measuring the absorbance at 620 nm (Thermo Scientific Multiskan FC, Waltham, MA, USA). The serial dilutions of glucose were used as a standard for the calibration curve. The results are expressed in mg/g of the sample FW.

### 2.4 Quantification of glucose

The modified method described by Khan et al. (2018) was used for glucose determination. The plant material (100 mg) was homogenized with 1 ml of extraction mixture containing 0.1 M phosphate buffer, 0.25 mg ortho-dianisidine dissolved in 1 ml methanol, peroxidase and glucose oxidase. The homogenized material was further centrifuged and the supernatant was heated in water bath at 35°C for 40 minutes. After incubation, the reaction was stopped by adding of 6 N HCl and the intensity of the pink colored reaction products was measured at 540 nm (Thermo Scientific Multiskan FC, Waltham, MA, USA). The glucose concentration in the plant samples corresponded to the intensity of the pink coloration. The serial dilutions of glucose, which were treated in the same way as the plant samples, were used as a standard for the calibration curve. The results are expressed in mg/g of the sample FW.

### 2.5 Quantification of fructose

For the determination of fructose, the modified of Messineo and Musarra (1972) was used. In brief, the plant material (50 mg) was homogenized in H_2_SO_4_ and then cysteine hydrochloride (2.5%) was added and vortexed. The homogenized material was further centrifuged and the supernatant was heated in water bath at 45°C for 10 minutes. During incubation, a green colored complex appeared. After cooling, tryptophan hydrochloride solution was added to the colored reaction products. The absorbance of the samples was measured after one hour at 518 nm (Thermo Scientific Multiskan FC, Waltham, MA, USA). The serial dilutions of fructose were used to generate the calibration curve. The results are expressed in mg/g of the sample FW.

### 2.6 Statistical analysis

All investigated parameters were analyzed in three biological samples at each time point. In addition, all measurements were repeated three times for each sample. The data were subjected to one-way analysis of variance (ANOVA) and the results are presented as mean ± SE. Comparisons between the mean values were performed using a Fisher LSD (the least significant difference) *post-hoc* test calculated at a confidence level of *p* ≤ 0.05.

## 3 Results

### 3.1 Spontaneous morphogenesis of *C. erythraea* in solid root culture

**Figure 1:**
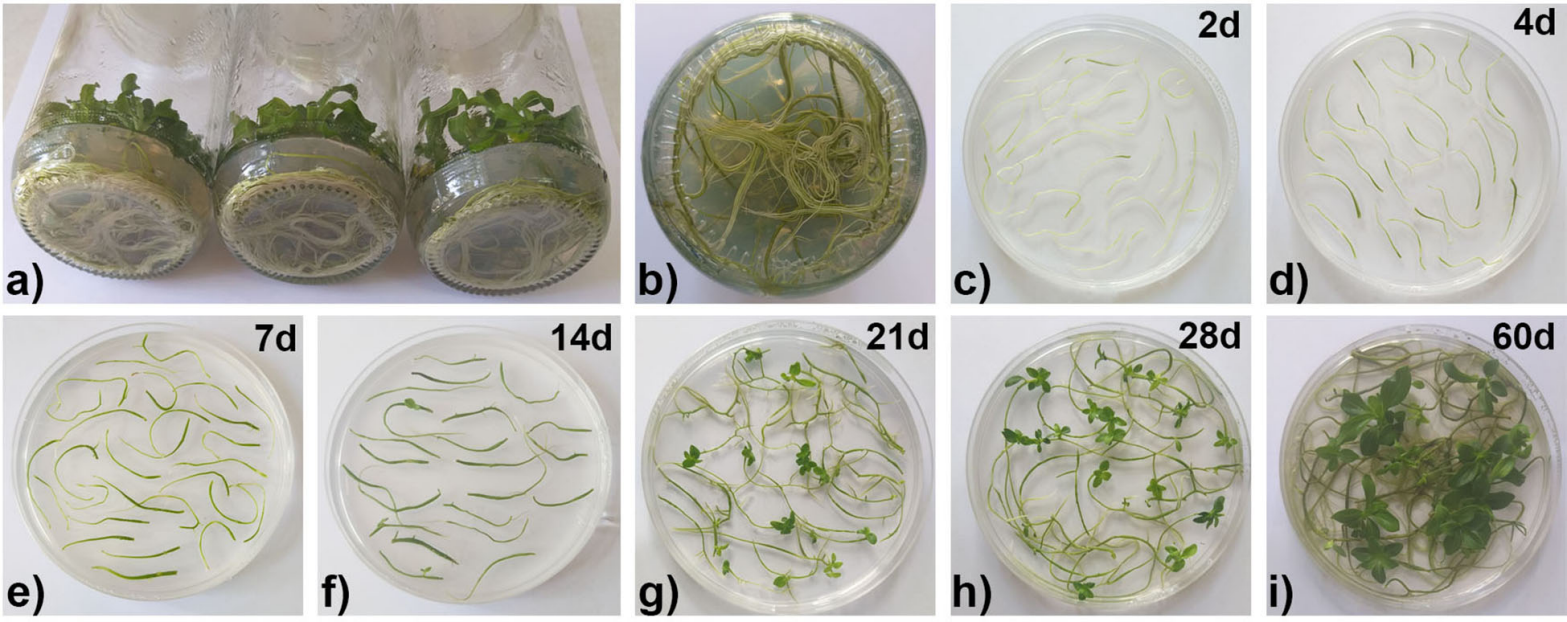
Induction of *in vitro* organogenesis in a solid root culture of *Centaurium erythraea*. Three-month-old mother stock cultures grown on ½MS hormone free medium **(A)**. Roots of *C. erythraea* used as primary plant material **(B)**. Cultured root explants after 2 days **(C)**, 4 days **(D)**, 7 days **(E)**, 14 days **(F)**, 21 days **(G)**, 28 days **(H)** and 60 days **(I)** on ½MS hormone free medium.

The roots of fully developed rosette centaury plants were used as primary plant material (**Figures 1A, B**). The solid root culture was established by placing the root tips (≈ 10 mm long) on hormone-free ½MS medium (**Figure 1C**). Shoot regeneration in the solid root culture of centaury occurred spontaneously. The first change in the cultured root explants was observed after only 4 days of cultivation (**Figure 1D**). The centaury roots grew thicker and thicker over the next 7 and 14 days (**Figures 1E, F**). The first regenerated adventitious buds were observed after 21 days (**Figure 1G**). Over the next few days, the root explants continued to elongate and thicken. After 28 days of culture, an increasing number of terminally regenerated shoots was observed (**Figure 1H**). Finally, after 60 days of culture, the fully developed centaury shoots with the typical rosettes were observed (**Figure 1I**).

### 3.2 Endogenous phytohormones in root explants of *C. erythraea*

**Figure 2:**
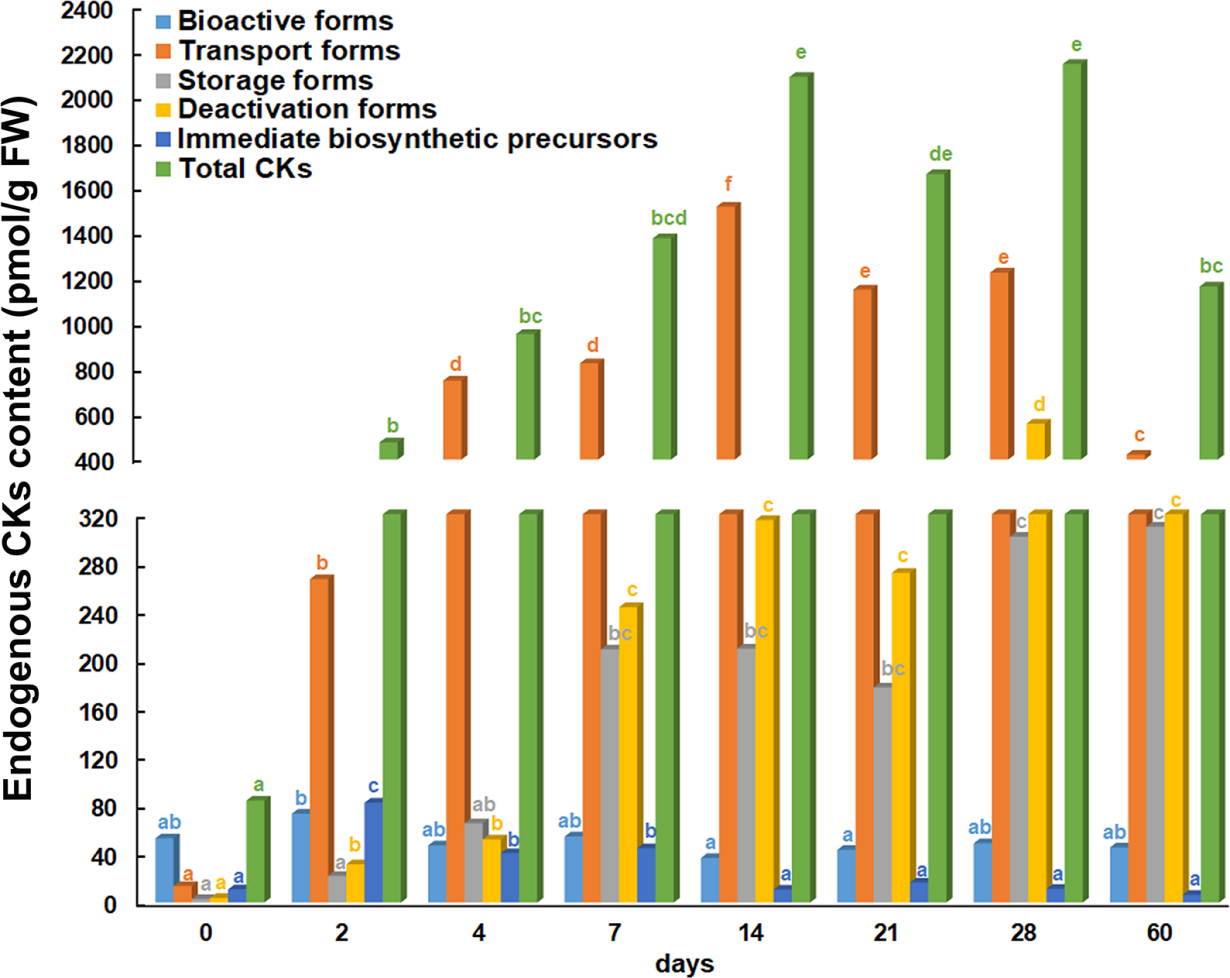
Endogenous cytokinin content in root explants of *Centaurium erythraea* after eight time points. Depending on physiological function and conjugation status cytokinins are categorised into five groups: bioactive, transport, storage, deactivated forms and immediate biosynthetic precursors. The data represent the mean values, while different letters indicate significant differences between time points (LSD test, *p* ≤ 0.05).

The endogenous phytohormones was quantified by LCMS method and the content of CKs in root explants after eight time points is shown in **Figure 2**. Five different groups of endogenous CKs were formed depending on physiological function and conjugation status as proposed by Lomin et al. (2015). The results presented showed that the bioactive CK forms did not vary significantly over time. Compared to the beginning of the experiment (day 0 – initial explant), bioactive forms increased only slightly after 2 days and decreased slightly after 14 and 21 days. The CK transport forms started to increase after 2 days and continued to increase after 4 and 7 days, while they reached the highest quantified values after 14 days. Thereafter, the transport forms started to decrease at day 21, 28 and 60. The content of storage forms started to increase after 4 days and continued this trend until the end of the experiment, when the highest values were determined (28 and 60 days). The deactivated CK forms also increased after 2 days, continued to increase after 7 days and remained at a similar level until the end of the experiment. The highest content of deactivated CK forms was observed after 28 days. Compared to the explants at the beginning of the experiment, the immediate biosynthetic precursors increased significantly at 2, 4 and 7 days, but from the 14^th^ and up to the 60^th^ day, the level of this group of CKs returned to the initial level. The lowest level of total CK content was found in the root explants at the beginning of the experiment. The first increase in total CK content was observed after 2 days. After 4 days of the experiment, the total CK content continued to increase until the 14^th^ day. After 21 days, it was observed that the total CKs content began to decrease until the end of the experiment, with the exception of the 28^th^ day, when a value similar to that after the 14^th^ day was recorded.

**Figure 3:**
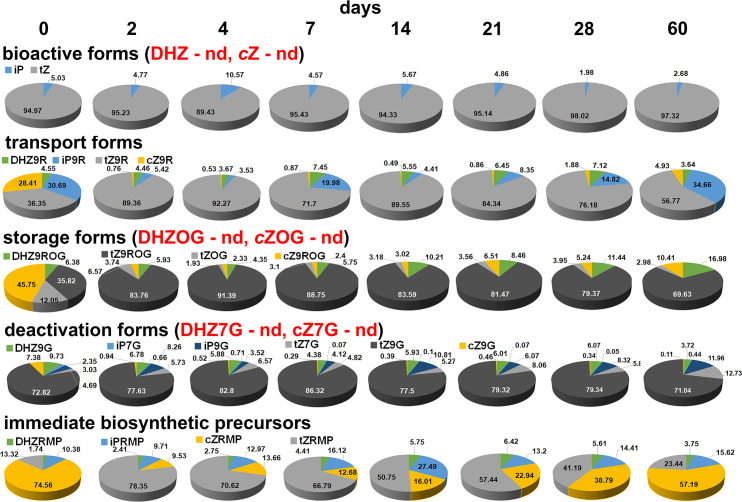
The proportion of individual metabolites within each cytokinin group in root explants of *Centaurium erythraea* after eight time points. Bioactive forms included DHZ, iP, *tZ*, *c*Z, transport forms included DHZ9R, iP9R, *t*Z9R and *c*Z9R, storage forms included DHZ9ROG, DHZOG, *t*Z9ROG, *t*ZOG, *c*Z9ROG and *c*ZOG, deactivated forms included DHZ7G, DHZ9G, iP7G, iP9G, *t*Z7G, *t*Z9G, *c*Z7G and *c*Z9G, immediate biosynthetic precursors included *t*ZRMP, DHZRMP, *c*ZRMP and iPRMP. nd, not detected.

Since the endogenous CKs were divided into five groups, it was very interesting to analyze the individual groups consisting of individual metabolites (**Figure 3**). The bioactive forms included DHZ, iP, *tZ* and *c*Z. In centaury root explants, DHZ and *c*Z were not detected at any of the eight time points. In all root samples examined at different time points, *tZ* was the major bioactive CK, accounting more than 90%, while the remainder was iP. All transport forms were detected, but their ratio changed at different time points. At the beginning of the experiment, the proportions of iP9R, *t*Z9R and *c*Z9R were approximately equal (≈ 30%) while the proportion of DHZ9R was 4.55%. After 2 days and until the end of the experiment, *t*Z9R represented the majority of the identified transport forms with a proportion of more than 50%. At several time points, including day 2, day 4, day 14 and day 21, a proportion of *t*Z9R of almost 90% was observed. At all eight time points analyzed, DHZ9R was quantified at a level of less than 10%, while *c*Z9R was quantified at a level of less than 5%. In addition, *c*Z9R was detected at almost trace levels, i.e. < 1%, at all time-points except the last. In the first and last points of the experiment, iP9R was determined approximately the same (≈ 30%). A slight decrease in iP9R was observed after 7 and 28 days. At all other time points, including 2, 4, 14 and 21 days, the percentage of iP9R was below 10%. The dynamic changes in iP9R content can be described as cyclical because it recursively decreased and increases. The storage forms included *O*-glucosides and 9-riboside *O*-glucosides. As with the bioactive forms, DHZOG and *c*ZOG were not detected in all samples analyzed. On the other hand, the proportion of successfully determined storage forms in centaury roots was similar after all eight time points, with one exception, day 0. At this time point, the ration of determined metabolites was as follows: *c*Z9ROG>*t*Z9ROG>*t*ZOG>DHZ9ROG. At all other time points, the content of single metabolite fluctuated slightly, but the same trend was observed. It was clearly established that *t*Z9ROG is the dominant metabolite with an average content of ≈ 80%. The proportion of the other metabolites detected can be represented as the following ratio: DHZ9ROG>*c*Z9ROG>*t*ZOG. The deactivated forms analyzed in this study comprised 7- and 9-glucosides. Certain metabolites such as DHZ7G and *c*Z7G were not detected in any sample. In general, *t*Z9G was the dominant metabolite in all analyzed centaury roots with a proportion of ≈ 80%. On the other hand, the lowest value of iP7G was determined in almost all samples. At the same time, the content of this metabolite was the highest even at the beginning of the experiment, while the content in all other samples was about ≈ 1.5 times lower. Similarly, the highest level of *c*Z9G was quantified in the initial root explants, while the level of this metabolite was several times lower at all other time points and even ≈ 70-fold lower after 60 days. The fifth group, the immediate biosynthetic precursors, included four riboside monophosphates. In the initial root explants, *c*ZRMP was the predominant metabolite. The proportion of this metabolite decreased significantly after 2, 4 and 7 days. After 14 days, the proportion of *c*ZRMP began to increase and persisted until the end of the experiment. In the initial explants, the levels of iPRMP and *t*ZRMP were approximately equal. After 2 days, the proportion of *t*ZRMP increased significantly to 78%, and from this point on, the proportion of *t*ZRMP began to decrease, with this metabolite being predominant at all time points. The riboside iPRMP maintained a similar proportion throughout the experimental period, except after 14 days when its highest content was detected. In all root explants analyzed, the DHZRMP content varied slightly, but was always detected in the smallest amount.

**Figure 4:**
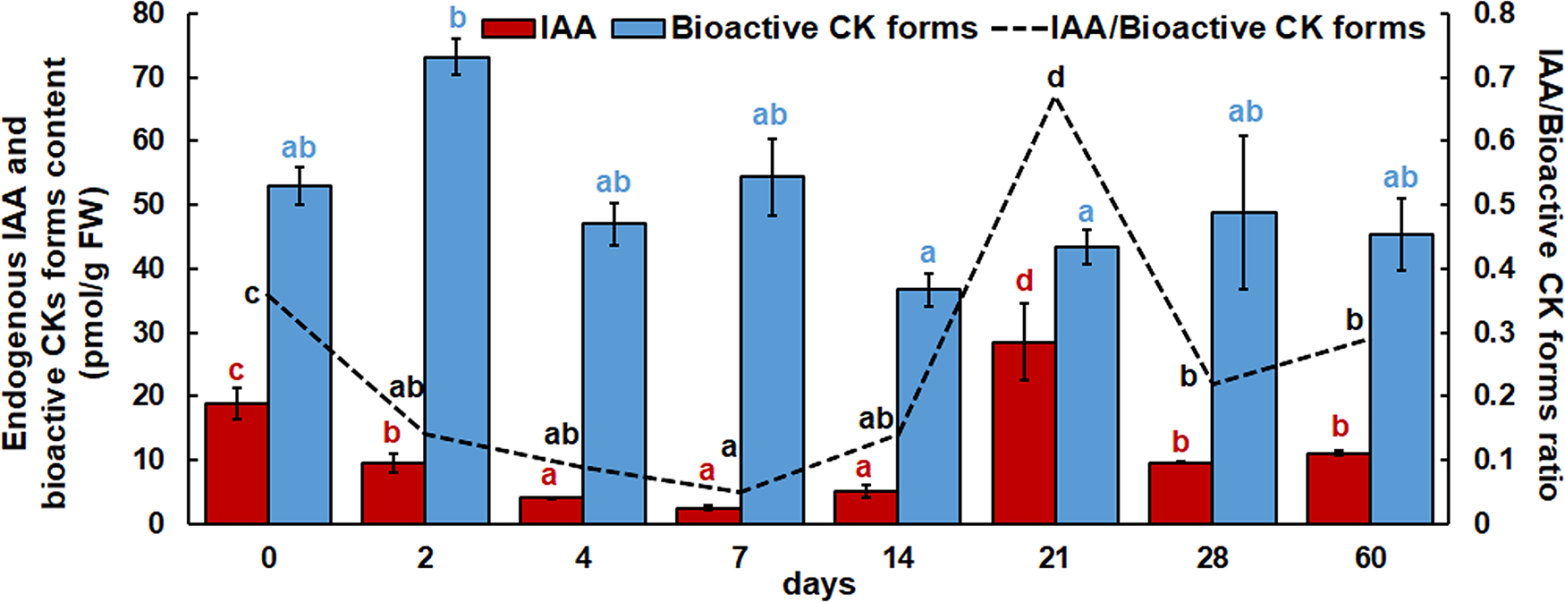
Endogenous IAA, bioactive cytokinin contents and their ratios in root explants of *Centaurium erythraea* after eight time points. The data represent mean values ± standard error. Different letters indicate significant differences between the time points (LSD test, *p* ≤ 0.05).

In addition to CKs, the endogenous IAA content was also quantified by LCMS method (**Figure 4**). Immediately after two days, IAA content decreased and continued to decrease after 4, 7 and 14 days. After 21 days, the IAA level increased significantly, and the maximum IAA content was found at this time point. After 28 and 60 days, the IAA concentration decreased again and returned to the values determined after two days. At the same time, the ratio of IAA/bioactive CK forms was examined. Compared to the beginning of the experiment, this ratio decreased after two days, remained unchanged until day 7 and then increased slightly after day14. After 21 days, however, a significant increase in the IAA/bioactive CK forms ratio was observed. After 28 and 60 days, the ratio decreased again.

Beside endogenous IAA content, the content of endogenous IAA conjugates was also determined (**Table 1**). The results obtained in this work showed that, IAA-Asp did not vary significantly during the whole experimental period, except for two time points, after 21 and 28 days. The same pattern was observed for another IAA conjugate, IAA-Glu. In contrast to the first two metabolites, IAA-GE increased significantly after 2 days, but continued to decrease until day 21, when a further increase was observed. The level of IAA-GE remained elevated until the end of the day 60. The maximum amount of the catabolite oxIAA was measured on day zero. Over the course of the 60-day experiment, oxIAA level decreased dramatically and varied slightly between time points. The content of oxIAA-GE and oxIAA-Asp did not change noticeably after the different time points. A reduced level of the catabolite oxIAA-GE was only observed after 4 days. On the other hand, the only significant increase in oxIAA-Asp was observed after 60 days. The content of the biosynthetic precursor I3A was the highest in the initial root explants. In all other samples tested, the content of this conjugate was significantly reduced.

**Table 1:** Content of endogenous IAA conjugates in root explants of *Centaurium erythraea* after eight time points.

time points	Irreversible amide-linked conjugates		Reversible ester-linked conjugate	IAA catabolites			Biosynthetic precursor
	IAA-aspartate	IAA-glutamate	IAA-glucose ester	2-oxindole-3-acetic acid	2-oxindole-3-acetic acid-glucose ester	2-oxindole-3-acetic acid-aspartate	indole-3-acetaldehyde
**0d**	0.58 ± 0.18^a^	0.96 ± 0.20^ab^	1.11 ± 0.17^a^	15.00 ± 2.02^c^	0.51 ± 0.18^c^	1.92 ± 0.23^ab^	11.71 ± 0.61^d^
**2d**	0.42 ± 0.11^a^	0.43 ± 0.13^a^	12.00 ± 0.30^c^	5.31 ± 0.51^ab^	0.38 ± 0.03^abc^	1.52 ± 0.23^a^	5.12 ± 1.03^abc^
**4d**	0.63 ± 0.35^a^	0.38 ± 0.07^a^	7.09 ± 0.94^ab^	4.38 ± 0.72^ab^	0.20 ± 0.04^a^	1.90 ± 0.24^ab^	3.93 ± 0.10^abc^
**7d**	0.83 ± 0.42^a^	0.57 ± 0.13^a^	8.48 ± 1.50^ab^	4.34 ± 0.95^ab^	0.32 ± 0.06^abc^	2.40 ± 0.20^ab^	3.71 ± 0.09^ab^
**14d**	0.60 ± 0.26^a^	0.52 ± 0.13^a^	5.46 ± 1.32^ab^	3.71 ± 0.40^a^	0.47 ± 0.13^bc^	1.70 ± 0.09^a^	5.73 ± 1.24^c^
**21d**	3.60 ± 0.51^b^	2.73 ± 0.44^c^	22.99 ± 2.65^d^	7.82 ± 1.63^b^	0.42 ± 0.05^abc^	3.39 ± 0.43^ab^	5,37 ± 0.38^bc^
**28d**	1.65 ± 0.15^a^	1.43 ± 0.07^b^	14.54 ± 3.64^c^	5.33 ± 1.76^ab^	0.30 ± 0.01^abc^	4.15 ± 0.18^b^	4.36 ± 0.42^abc^
**60d**	0.59 ± 0.42^a^	0.44 ± 0.11^a^	25.18 ± 1.83^d^	3.84 ± 0.59^a^	0.24 ± 0.01^ab^	8.40 ± 2.21^c^	3.35 ± 0.19^a^

The amount of each conjugate is given in pmol/g FW. The data represent mean values ± standard error. Different letters indicate significant differences between the time points (LSD test, *p* ≤ 0.05). IAA-Asp, IAA-aspartate; IAA-Glu, IAA-glutamate; IAA-GE, IAA-glucose ester; oxIAA, 2-oxindole-3-acetic acid; oxIAA-GE, 2-oxindole-3-acetic acid-glucose ester; oxIAA-Asp, 2-oxindole-3-acetic acid-aspartate; I3A, indole-3-acetaldehyde.

### 3.3 Sugar concentration in root explants of *C. erythraea*

**Figure 5:**
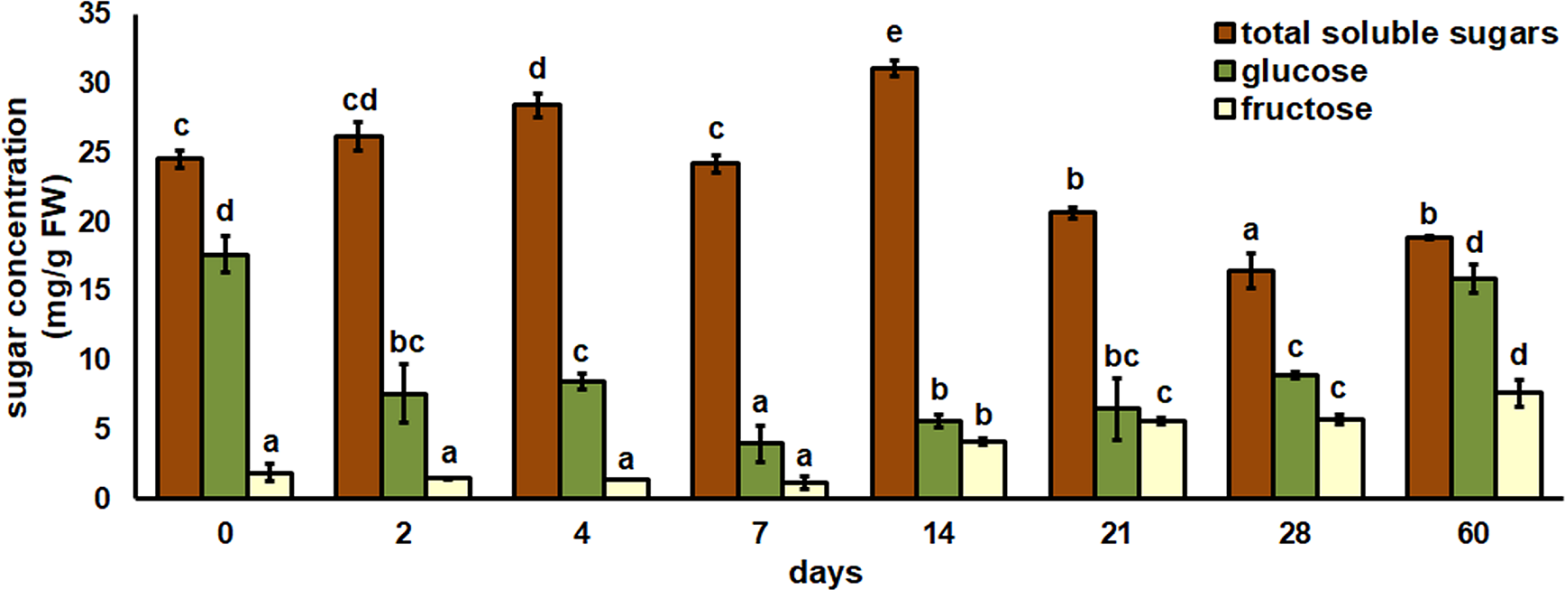
Total soluble sugar, glucose, and fructose concentrations in root explants of *Centaurium erythraea* after eight different time points. Data represent mean values ± standard error. Different letters indicate significant differences between the time points (LSD test, *p* ≤ 0.05).

The total content of soluble sugars (TSS), glucose and fructose was determined in root explants after eight different time points (**Figure 5**). The slight increase in TSS was observed immediately after two days. The first significant increase in TSS was found after 4 days, and after 7 days TSS returned to the control level. The highest TSS level was observed after 14 days. The last three time points of the experiment showed that the TSS concentration decreased significantly to the control. The highest glucose concentration was found at two time points – in the initial root explants and after 60 days of cultivation. The glucose concentration decreased significantly after 2 and 4 days and continued to decrease after 7 days. After 14, 21 and 28 days, an increase in glucose concentration was observed but it was still significantly lower than the control value. The fructose concentration did not change after 2, 4 and 7 days compared to the control. The first significant increase in fructose was observed after 14 days, from then on fructose continued to increase until the last time point of the experiment, when the highest concentration was observed.

## 4 Discussion

A large number of factors and their interactions are involved in the morphogenesis of plants. Only a limited number of species naturally regenerate the whole plantlets after cutting. A review of existing and available literature data revealed that the first studies focused on the analysis of morphogenetic processes only after exogenous application of plant growth regulators and nutrients, mainly sugars, in tissue culture media. In recent years, the approach has fundamentally changed and numerous endogenous factors are intensively studied. Accordingly, the assessment of hormone profiles at different time points during regeneration can lead to a better understanding of the process of spontaneous morphogenesis of centaury and contribute to the understanding of this phenomenon in general.

The maximum of total CKs accumulation was observed in centaury root explants from the 14^th^ to the 28^th^ day of cultivation. Since the explants began to thicken significantly after 14 days and the first regenerated adventitious shoots appeared after 21 days, it can be concluded that CK accumulation was accompanied by intense cell divisions. This certainly confirms the assertion of Werner et al. (2021), who described high CK content in young vegetative and reproductive organs progressing during the cell division phase. In the current study, the free CK base *t*Z was found to be the dominant CK in centaury root explants, while *c*Z and DHZ were not detected. This result suggests that the amount and structure of cellular CKs depend on biosynthesis, transport, and inter-conversions of specific forms. Interestingly, *t*Z was detected at about 90% at all time points examined, while the remainder was iP. It is known that *tZ* is produces from iP, and these two bioactive CKs differ, only in hydroxylation of the side chain (Kiba et al., 2023). This result suggests that the most of the synthesized iP was converted to *tZ*. Although the main site of *t*Z biosynthesis is the root vasculature, from where it is transported to the shoot via xylem, it has been shown that *tZ* might stimulate and enhance shoot growth (Kiba et al., 2013). Considering that *tZ* was the major CKs in initial root explants isolated from rooted centaury shoots, but also in all other time points tested, it can be concluded that *tZ* is equally present during shoot development in intact plants, but also during shoot regeneration from isolated root explants.

Cytokinin ribosides are intermediates between highly the active CK free bases and the inactive nucleotide precursors and traditionally considered mainly transport forms (Nguyen et al., 2021). In the current study, the transport CK forms started to increase after 2 days and reached the maximum values on the day 14, but thereafter they decreased until the end of the experiment. Even after 2 days and until the end of the experiment, *t*Z9R represented the majority of the identified transport forms, accounting for more than 50%. This finding is in accordance with our previous work, where we showed that in roots of 4-week-old centaury plants, *t*Z9R was the predominant CKs root riboside (Trifunović-Momčilov et al., 2016). The presence of *t*Z9R is very important as this CK form is acropetally transported in xylem sap (Skalický et al., 2018). The results presented strongly suggest that ribosides are responsible for developmental changes and represent a very important factor in the regulation of physiological traits, which has already been described by Nguyen et al. (2021).

The storage forms are formed after reversible *O*-glucosylation of the side chain of the CK molecule. Besides, when glucose is attached to the *N*7 or *N*9 atoms of the purine ring, the CKs form irreversible *N*-glucosides, which are biologically stable and are among the most common naturally occurring CK forms (Vylíčilová et al., 2020; Pokorná et al., 2021). These two types of glucose conjugates play different roles in protecting CKs from degradation and inactivation. In the current study, *t*Z9ROG was shown to be the predominant metabolite (≈ 80%) in initial root explants. Considering that *t*Z was very abundant during shoot regeneration from root explants at all time points studied, the content of *t*Z9ROG indicates an active and successful conversion from the storage form to the bioactive form and vice versa. The results obtained are consistent with the claims of Hluska et al. (2021) who reported that, when CKs are required to a greater extent, they are usually hydrolysed and further transported to the subcellular compartments. In contrast, when CKs are no longer required, they are glucosylated and transported to the vacuole, where they are stored as inactivation products. Almost 40 years ago, *N*-glucosides were considered active CKs, but when it was shown that they do not bind to CK receptors, they were classified as inactive forms (Hluska et al., 2021). Normally, 7- and 9-glucosides are resistant to glucosidase and cannot be converted into active CK forms. Glucosyltransferases catalyze the formation of both *N*-glucosides with the observation that the hydrogen atom attaches to the *N*7 position of the adenine molecule, resulting in higher levels of the 7-glucosides during in assays *in vitro* (Hou et al., 2004). This may indicate that glucosyltransferases, produce higher amounts of 7-glucosides in aqueous solutions. The results of this work showed that some 7-glucosides, including DHZ7G and *c*Z7G, were not determined in any of the samples analysed, while iP7G was detected in all samples, but at the lowest level. In fact, 9-glucosides were the predominant form in all centaury roots. The observed differences between the accumulation of 7- and 9-glucosides may be attributed to different tissue-specific activities towards certain N-glucosides or even to the different activity of the enzymes responsible for hydrolysis and not to the activity of glucosyltransferases, which seem to act non-specifically as recently suggested (Hoyerová and Hošek, 2020).

During the final steps of CK biosynthesis, the monophosphates can be directly converted to their respective active free bases in one-step by a specific phosphoribohydrolase encoded by a gene lonely guy – LOG (Hluska et al., 2021). The LOG reaction is fast and effective, and immediately generates active free bases without riboside formation. In addition to the LOG reaction, there is also a two-step process, in which riboside forms are first formed, and can then be converted into free base forms (Nguyen et al., 2021). In the present study, all four riboside monophosphates (DHZRMP, iPRMP, *c*ZRMP, and *t*ZRMP), were detected in centaury root explants. In the first and last time points of the experiment, *c*ZRMP was the predominant monophosphate, while in the other time points *t*ZRMP predominated. The other two monophosphates maintained a similar proportion throughout the experimental period, while DHZRMP was detected in the lowest amount. All of these CK precursors are intrinsically inactive, and must be converted to free bases in order to have a hormonal effect. The detection of all monophosphates in centaury root explants may indicate that there is a different need for effective free base production or that riboside production must be avoided, which is difficult to conclude. There is evidence that riboside intermediates are only produced when the plant needs to transfer the CKs signal from the source of biosynthesis, and further LOG reaction is activated after the arrival of the signaling molecule in the target organ/tissue, where active free bases are produced (Nguyen et al., 2021). It is very likely that the two-step process involving ribosides as immediate biosynthetic precursors only occurs when either remote signaling and/or local CK pooling is required before active CK forms are actually needed, which has already been postulated by Nguyen et al. (2021). As already previously stated as initial root explants, the three-month-old mother stock cultures were used. The initial root explants originated from centaury shoots grown and rooted *in vitro*. It is quite possible and also expected that centaury roots have a fairly stable and balanced content of endogenous hormones during usual growth and development *in vitro*. When root tips were isolated and placed individually on ½MS medium, they start to have their own development pathway. In that regard, quick and the most dramatic changes of single CK metabolites, especially transport and storage forms as well monophosphates, during the first two days is not surprising at all, and is quite expected.

As with CKs, endogenous cellular auxin concentration depends on *de novo* synthesis, catabolism and transport. The most bioactive form of auxin in plants is IAA. The determination of endogenous hormones during spontaneous morphogenesis of centaury revealed that the IAA concentration started to decrease after only two days of the experiment and decreased continuously until the day 21, when a significant increase was detected. The similar results were observed during the induction and development of protocorm-like bodies of *Cattleya tigrina*, when the authors linked the decrease in endogenous IAA content to intense cell division and differentiation (De Conti et al., 2018). As previously mentioned, the significantly increased IAA content in centaury root explants was detected on day 21, when the first regenerated adventitious shoots were noticed. This result can be explained as a specific morphogenic response of centaury root explant. Examination of hormone levels in the bud meristem of *Phalaenopsis* also showed that high concentrations endogenous IAA induced cell elongation and division, with all subsequent outcomes for plant growth and development (Zhang et al., 2019). This is also supported by the fact that during *in vitro* organogenesis of wheat, the cells forming the root meristem were strongly immune-stained strongly for IAA, but the intensity of immunostaining decreased with increasing distance from the root tip (Seldimirova et al., 2016). The balance between auxin and CKs is very important. According to Ikeuchi et al. (2019), the auxin/CKs ratio determines the fate of the regenerating organs – a high auxin/CKs ratio leads to root regeneration, while lower auxin/CKs ratio triggers shoot regeneration. In centaury root explants, the ratio of IAA/bioactive CK forms was examined, and the results showed that the ratio decreased after two days and continued until day 21, when a significant jump in the ratio of IAA/bioactive forms ratio observed. A similar pattern was observed in shoot regeneration from pineapple leaf explants, where the lowest value of the ratio between endogenous IAA and CKs was recorded on the third day, suggesting that the first early days of the experiment represent the induction phase of shoot formation (Mercier et al., 2003). That the relationship between CKs and auxin is very important during *in vitro* organogenesis was also shown by Hu et al. (2017), who hypothesized that CKs represent the most important factor for shoot organogenesis and auxin inhibits the action of CKs. The same authors postulated that the recalcitrance of numerous plant species during *in vitro* organogenesis could be partly due to relatively high concentrations of endogenous auxin. The results presented in this paper showed a higher content of bioactive CK forms compared to endogenous IAA content. This trend was observed throughout the experimental period, with the exception of one time point – day 21 when the IAA content increased significantly, and at the same time the ratio between IAA and bioactive CK forms ratio increased significantly, triggering the centaury shoot regeneration.

Maintaining a homeostatic level of active auxin in plant tissues is crucial for plant growth and development. The results obtained in this work showed that the levels of two analyzed irreversible amide-bound conjugates, IAA-Asp and IAA-Glu, did not vary notably, except after day 21 when a significant increase was detected. At the same time point of the present experiment, a significantly increased IAA content was observed. Nevertheless, the level of detected amide-linked conjugates was much lower in comparison to the IAA content. This result may indicate that IAA-Asp and IAA-Glu may serve as storage forms that can be easily converted into active IAA, as recently shown (Hayashi et al., 2021). The increased level of the reversible ester-bound conjugate IAA-GE was determined after 2 days and from day 21 until the end of the experimental period. Moreover, the level of free IAA in centaury roots was similar to the level of free IAA-GE, a reversible glucose conjugate, that normally serves as an auxin storage form to maintain the metabolic balance of auxin (Akabane et al., 2024). The levels of catabolites, including oxIAA, oxIAA-GE and oxIAA-Asp, varied slightly between the different time points. Interestingly, both the catabolite oxIAA and the biosynthetic precursor I3A were determined to be highest in the initial centaury root explants, but their levels continued to decrease significantly. One of the major IAA inactivation pathways is the oxIAA pathway, in which dioxygenase catalyzes the oxidation of IAA to oxIAA (Zhang and Peer, 2017). The reduced oxIAA content in centaury root explants may be attributed to increased auxin action, as the IAA oxidation pathway responds much slower to IAA, compared to the specific GRETCHEN HAGEN3 amido synthetases, which also conjugate IAA to amino acids, but can still be modulated by slight changes in IAA levels (Stepanova and Alonso, 2016). On the other hand, in addition to indole-3-acetamide and tryptamine, I3A has also been proposed as an IAA precursor, although the role of these three metabolites in the IAA biosynthetic pathway is still unclear (Kasahara, 2016). The reduced I3A content in the centaury roots is not so surprising, as it was recently found that the indole-3-acetamide pathway is not always essential for the overall supply of IAA to the plant (Pérez-Alonso et al., 2021).

Previous studies have confirmed that endogenous carbohydrates are a key factor in the formation of underground storage organs such as bulbs, rhizomes and tubers (Marković et al., 2020; Morales-Fernández et al., 2015; Ren et al., 2021; Xu et al., 2020; Tejeda-Sartorius et al., 2022). The important role of soluble sugars was also discovered during the process of somatic embryogenesis in *Cyathea delgadii* (Grzyb et al., 2017), *Begonia tuberous* (Khai et al., 2021) and *Carica papaya* (Vale et al., 2018). Nevertheless, the role of endogenous sugars in different time points of morphogenesis is not clear enough. The use of carbohydrates in plant tissue culture primarily implies the exogenous addition of sucrose to the culture medium, which is a fuel source for optimal development and maintenance of osmotic potential (Gago et al., 2014). In centaury root explants, total soluble sugars (TSS) began to increase after two days and continued to increase after four days. The strong increase was also observed after two days during organogenesis of *Cedrela fissilis* (Aragão et al., 2016) and two days after induction of protocorm-like bodies from leaf explants of *C. tigrina* (De Conti et al., 2018). This high endogenous TSS content indicates that the first days of culture are an important period for the uptake of exogenous sucrose from the culture medium. Considering that the first changes in cultured centaury root explants were observed after 4 days of cultivation, carbohydrate uptake from the culture medium at the beginning of cultivation may be associated with the promotion of explant growth. As the cultivation progressed, the centaury root explants thickened and the maximum TSS content was determined after 14 days. This observation can be explained by the accumulation of sugars for upcoming regeneration processes. Considering that the first regenerated adventitious buds were appeared after 21 days, when a significant decrease in TSS content was observed, it can be assumed that this is the key point for the increased energy demand. After 21 days, the TSS content continued to decrease while, at the same time, an increased number of regenerated shoots was observed, which could indicate a prolonged energy demand. Glucose and fructose, the reducing sugars, are the main hydrolysis products of sucrose (Patrick et al., 2013). During the present experiment, the glucose concentration fluctuated at during different time points, and the highest glucose concentration was observed in the initial root explants and at the last time point examined. At the same time point, on day 60, the highest fructose concentration was also found. This result could indicate that at this time sucrose was intensively absorbed from the culture medium and further metabolized into glucose and fructose, which is crucial for the further growth of regenerated shoots. A similar result was obtained at the end of the culture period in shoot propagation from cotyledonary nodal segments of *C. fissilis*, when the concentration of reducing sugars was increased (Aragão et al., 2016). On the other hand, the reduced concentrations of glucose and fructose at every time point before the end of experiment, can be primarily explained by the basic carbohydrate metabolism of centaury root explants. This result is consistent with previous findings obtained in *Tulipa edulis* during stolon development into a new bulb (Miao et al., 2016). Accordingly, it is evident that the fluctuations in glucose and fructose concentrations indicate that these sugars are coordinated during spontaneous morphogenesis of centaury.

During morphogenesis and plant development both CKs and carbohydrates play very important individual but also combined roles. Still, the crosstalk between these two regulators has not been systematically investigated. The available literature data indicate very complex carbohydrates/CKs interactions dependent on the plant organ and ongoing physiological process (Wang et al., 2021). Based on the results obtained on *Arabidopsis* seedlings it was shown that glucose and CKs interplay can be antagonistic or even agonistic on gene expression while, at the same time, glucose had a strong effect on genes involved in CKs metabolism and signaling (Kushwah and Laxmi, 2014). The elucidating of interaction between carbohydrates and CKs can be very beneficial in understanding the mechanisms and regulatory networks involved in numerous developmental processes throughout plant life.

## 5 Conclusion

The aim of this study was to analyze the dynamic changes in the content of endogenous phytohormones and carbohydrates in root explants at different time points during spontaneous centaury morphogenesis *in vitro*. To our knowledge, this is the first report on the endogenous hormone profile and simultaneously on the effects of endogenous carbohydrate content during spontaneous centaury morphogenesis *in vitro*. Based on the results obtained, we noticed that *tZ* is the most abundant during shoot regeneration on isolated root explants. Moreover, a significant increase in IAA content was observed after 21 days in culture, when the first regenerated adventitious shoots appeared. At the same time point, a tremendous increase in the ratio of IAA to bioactive CKs was detected, implying that an increase in IAA content significantly altered the IAA/CKs ratio, which was beneficial for shoot regeneration. Furthermore, the highest TSS content detected was after 14 days, indicating the importance of sugar accumulation for the subsequent regeneration of adventitious shoots, which was detected one week later. The most dramatic changes in endogenous bioactive CKs, IAA and carbohydrate homeostasis were found to occur in the period from day 14 to day 21, which is undoubtedly a valuable and crucial time for the onset of shoot regeneration. The process of spontaneous centaury morphogenesis *in vitro* is a very interesting phenomenon that should definitely be investigated further. Accordingly, future research at the molecular level is expected to provide new information that may contribute to a better understanding of the spontaneous morphogenesis pathway, a developmental process specific to *in vitro* cultured centaury root explants.

## Data Availability

The raw data supporting the conclusions of this article will be made available by the authors, without undue reservation.
